# An Anti-hyperventilation Instruction Decreases the Drop in End-tidal CO_2_ and Symptoms of Hyperventilation During Breathing at 0.1 Hz

**DOI:** 10.1007/s10484-019-09438-y

**Published:** 2019-05-07

**Authors:** Mikołaj Tytus Szulczewski

**Affiliations:** Faculty of Psychology, University of Warsaw, Stawki 5/7, 00-183, Warsaw, Poland

**Keywords:** Slow breathing, Paced breathing, Breathing at 0.1 Hz, Hyperventilation, Partial pressure of end-tidal carbon dioxide, PetCO_2_

## Abstract

Breathing at a frequency of around 0.1 Hz is widely used in basic research and in applied psychophysiology because it strongly increases fluctuations in the cardiovascular system and affects psychological functioning. Volitional control of breathing often leads to hyperventilation among untrained individuals, which may produce aversive symptoms and alter the psychological and physiological effects of the paced breathing. The present study investigated the effectiveness of a brief anti-hyperventilation instruction during paced breathing at a frequency of 0.1 Hz. Forty-six participants were randomly assigned to one of two groups: a group given an anti-hyperventilation instruction and a control group without such an instruction. The instruction asked participants to avoid excessively deep breathing and to breathe shallowly and naturally. Participants performed the breathing task for 10 min. Hyperventilation was measured by partial pressure of end-tidal CO_2_ (PetCO_2_); furthermore, symptoms of hyperventilation, feeling of air hunger, task difficulty, and affective state were measured by self-report. The results showed that paced breathing without instruction decreased PetCO_2_ by 5.21 mmHg and that the use of the anti-hyperventilation instruction reduced the drop in PetCO_2_ to 2.7 mmHg. Symptoms of hyperventilation were lower in the group with the anti-hyperventilation instruction. Neither the feeling of air hunger nor task difficulty were affected by the instruction. There were no significant effects of the instruction on affective state. The present study indicates that a brief anti-hyperventilation instruction may be used to decrease drop in PetCO_2_ and symptoms of hyperventilation during breathing at 0.1 Hz and that the instruction is well tolerated.

Breathing paced by a visual or aural cue (paced breathing) is widely used in basic research, applied psychology, and behavioral medicine. The most widely studied and used paced breathing is at frequencies around 0.1 Hz (six breaths per minute) because this frequency maximizes the amplitude of cardiovascular-oscillations (Pitzalis et al. 1998; Song and Lehrer 2003). Breathing at 0.1 Hz has been used as an emotion regulation tool (Laborde et al. 2016; Zautra et al. 2010), as an experimental procedure in basic research on breathing (Chalaye et al. 2009; Szulczewski and Rynkiewicz 2018), as a method of measurement of vagal tone (Grossman et al. 1990; Shields 2009), and as a behavioral treatment for hypertension (Zou et al. 2017). Breathing at frequencies around 0.1 Hz is also used in biofeedback-based methods aimed at increasing heart rate variability (Lehrer et al. 2000), which has shown promising effects in the treatment of, for example, anxiety (Goessl et al. 2017), depression (Hassett et al. 2007; Siepmann et al. 2008), and fibromyalgia (Hassett 2007). Furthermore, several mobile applications have recently been developed that attempt to reduce stress using slow paced breathing (e.g. Paced Breathing, Trex LLC).

Paced breathing may lead to hyperventilation, which is not desirable in either basic research or applied psychophysiology. The medullary homeostatic mechanism, using information from chemoreceptors (CO_*2*_, pH) plays a key role in the regulation of spontaneous breathing (Del Negro et al. 2018). During paced breathing, neural control of respiration is shifted to cortical areas (Mckay et al. 2003; Šmejkal et al. 2000). A shift in neural control may decrease the contribution of medullary homeostatic control and lead to excessive ventilation. Moreover, slow breathing increases the amplitude of arterial gas oscillations, which may increase respiratory drive (Semple 1984). Paced breathing also drastically reduces variability in breathing pattern, decreasing flexibility of respiratory responses to sudden changes. For this reason, as an anticipatory prevention of hypoventilation (during a sudden increase in respiratory demands), the organism may increase ventilation above its actual needs. Furthermore, paced breathing prevents compensatory mechanisms such as sighing and apnoea. As a consequence, some participants tend to experience hyperventilation during slow paced breathing, which results in the removal of an excessive amount of CO2 from the blood (Anderson et al. 2009; Szulczewski and Rynkiewicz 2018).

When hyperventilation is strong enough, it results in respiratory alkalosis which produces symptoms such as paresthesia, tetany, headache, dizziness, among others (Hornsveld et al. 1990). Hyperventilation also affects the cardiovascular system, which is often the target of paced breathing tasks in applied psychophysiology and basic research. Hyperventilation increases heart rate, decreases mean arterial pressure (Bharucha et al. 1996; Ford et al. 1995), and decreases respiratory sinus arrhythmia (George et al. 1989; Henry et al. 1998; Sasano et al. 2002). Furthermore, studies on the etiology of panic attacks have shown that hyperventilation-related physiological changes may influence affective arousal (Carter et al. 2001; Meuret et al. 2011; Thyer et al. 1984; Wilhelm et al. 2001; Van de Borne et al. 2000). Therefore, hyperventilation may modify the results of basic research that employs paced breathing as well as the effectiveness of applied methods that use slow paced breathing. Study of heart rate variability biofeedback suggests that after longer training under appropriate guidance, the tendency to hyperventilate diminishes (Vaschillo et al. 2006). Similarly, respiration training aimed at decreasing hyperventilation using breathing exercises with capnometry biofeedback has proven to be effective at decreasing hyperventilation among individuals suffering from panic disorder and asthma (Kim et al. 2012; Meuret et al. 2018; Ritz et al. 2014). However, hyperventilation may still be of prominent in research that uses paced breathing without training as well as when paced breathing is performed without sufficient guidance, for example when it is used as a self-help method.

The simplest way to avoid hyperventilation during paced breathing tasks is an instruction to avoid deep breathing and breathe shallowly. However, little research has been conducted on the effectiveness of anti-hyperventilatory instructions without capnometry biofeedback. A relatively long (12 min) video-based anti-hyperventilation instruction has previously been used as a brief intervention against phobia and it proved effective at increasing partial pressure of CO_2_ at the end of exhalation (PetCO_2_) during a phobia elicitvation procedure (Meuret et al. 2017). A study by Conrad et al. (2007) suggests that shorter instructions that could be easily implemented in basic research might be ineffective. In this study, the effects of a short instruction to breathe shallowly and slowly were investigated. The instruction increased negative affect and was not effective at changing respiratory rate or decreasing PetCO_2_. Given that respiratory rate was not paced in either of the aforementioned studies, the results cannot be directly extrapolated to paced breathing tasks.

The present study aimed to investigate the effectiveness of a brief anti-hyperventilation instruction in reducing PetCO_2_ and symptoms of hyperventilation during breathing at 0.1 Hz. Perceived task difficulty was measured to examine whether the anti-hyperventilation instruction influenced the difficulty of the paced breathing task. Furthermore, the effects of the instruction on the feeling of air hunger were investigated, as this dyspnoeic sensation is produced by hypoventilation, therefore it may be increased by an instruction aimed at decreasing ventilation. To examine the effects of the instruction on the affective consequences of paced breathing, affective state was measured before and after the breathing task. In order to investigate whether the drop in PetCO_2_ during breathing at 0.1 Hz has affective consequences, the present study also examined the relationship between changes in PetCO_2_ and changes in affective state.

## Method

### Participants and design

Forty-six participants took part in the study (23 women and 23 men). Participants were recruited by an announcement to a group of students from the University of Warsaw via social media. The age of the participants ranged from 19 to 26 (*M *= 21.88, *SD *=1.71). Exclusion criteria were self-reported diseases of the respiratory system (e.g. asthma), psychiatric and neurological diseases, or chronic diseases (e.g. diabetes). Participants were asked to refrain from physical exercise, caffeinated beverages, and smoking for 3 h before the study. All participants signed a written consent form and were informed that they can resign from the study at any time. Participants received the equivalent of approximately 7 euros for participating in the study. Approval from the local ethics committee was obtained (the ethical committee of the University of Warsaw). Participants were assigned to groups based on an ordering prepared before the study using the random number generator function in Microsoft Excel (2007). In the experimental group, breathing at 0.1 Hz was preceded by an anti-hyperventilation instruction while in the control group breathing at 0.1 Hz was not preceded by an anti-hyperventilation instruction. After baseline measurement, participants performed a 10-minute-long paced breathing task. Affective state, symptoms of hyperventilation, and feeling of air hunger were measured before and after the paced breathing task. The difficulty of paced breathing was measured after the task.

### Apparatus and materials

Symptoms of hyperventilation were measured with a 0–6 Likert scale ranging from zero (not experiencing any symptoms at all) to six (experiencing symptoms with maximal imaginable intensity). The following symptoms of hyperventilation were examined: dizziness, tingling and pricking, numbness, headache, and increased muscle tension in hands and feet. These symptoms were drawn from the Nijmegen Questionnaire (Van Dixhoorn and Duivenvoorden 1985) and a study by (Hornsveld et al. 1990). The mean of all symptoms was computed for further analysis. The scale obtained on the basis of all symptoms had high internal consistency (Cronbach’s α = 0.85). Participants were also asked to rate the difficulty of the task on a 0–6 scale (from extremely easy to extremely difficult). Air hunger sensation was measured with a 0–6 Likert scale ranging from zero (not experiencing air hunger at all) to six (experiencing air hunger with maximal imaginable intensity). Affective state was assessed with the Polish translation of the Two-Dimensional Mood Scale (Sakairi et al. 2013), a tool based on the two dimensional model of affect (Barrett et al. 2007; Russell 2003). This model assumes that there are two basic dimensions of affect: arousal and valence. Furthermore, when rotated by 45°, its axes give separate measures of arousal with pleasant valence and arousal with unpleasant valence (Yik et al. 1999; Yik et al. 2011). In the present study we used four measures of affective state: general arousal and valence; and two measures obtained from rotation of the basic dimensions by 45° – pleasant arousal (the vitality scale in the questionnaire) and unpleasant arousal (reversed stability scale in the questionnaire). Indices were computed with the use of equations described by Sakairi et al. (2013). Scales had acceptable internal consistency: high arousal-pleasure (Cronbach’s α = 0.79), low arousal-displeasure (Cronbach’s α = 0.67), low arousal-pleasure (Cronbach’s α = 0.76), and high arousal-displeasure (Cronbach’s α = 0.77).

PetCO_2_ and respiratory rate were measured with an infrared sidestream capnometer (Capnocheck Plus, model 9004-000, BCI International, Waukesha, WI, USA) by nasal cannula. PetCO_2_ was recorded in millimeters of mercury (mmHg) for 4-second periods and exported to a PC computer. Mean PetCO_2_ and respiratory rate were computed for the baseline period and paced breathing task in MATLAB (2014, The MathWorks, Natick, MA, USA).

Due to a failure, capnometer respiratory data from one participant was not recorded. Due to an error in experimental procedure, self-reported data from six participants were not saved. One participant was excluded from analysis because she reported feeling unsafe in the laboratory cabin due to claustrophobia and did not change her breathing frequency properly.

### Procedure

Participants signed the consent form. Then, participants were seated in front of a PC computer and the nasal cannula was put on. The experiment was run using OpenSesame 3.0.7 software (Mathôt et al. 2012). The experiment began with a baseline period which lasted 6 min. Participants sat with their eyes open and were asked to breathe only through the nose. The first minute of baseline measurement was an adaptation period and the subsequent 5 min constituted the baseline period which was used in the analysis. Then participants answered questions about affective state, air hunger, and symptoms of hyperventilation. Next, participants received either the anti-hyperventilation instruction or no instruction (in the control group) and performed a 10-minute-long paced breathing task at 0.1 Hz. Breathing was guided by a sound cue, with inhalation lasting four seconds and exhalation lasting six seconds. This ratio was used because a study by Wang et al. (2013) showed that participants tend to have longer exhalation during breathing at 0.1 Hz when length of breathing phases is not enforced. This ratio also induces a greater decrease in affective arousal (Cappo and Holmes 1984; van Diest et al. 2014). The experimenter monitored the capnometer measurement to make sure that the participants were performing the paced breathing task. After the paced breathing task, participants once more answered questions about their current affective state and retrospectively about air hunger and symptoms of hyperventilation during paced breathing and the perceived difficulty of the task.

### Anti-hyperventilation instruction

The anti-hyperventilation instruction was presented in text form on the computer just before the paced breathing task. The instruction was as follows: “In order to avoid hyperventilation during the paced breathing task, please avoid excessively deep breathing. Breathe shallowly and naturally.” The instruction was based on an instruction used in heart rate variability biofeedback (Lehrer et al. 2000).

### Data analysis

Statistical analyses were performed in IBM SPSS Statistics for Windows (version 22.0, IBM Corp., Armonk, NY). The significance threshold was set at *p* < 0.05. To see if participants had changed their breathing frequency, respiratory rate was examined using a repeated measures ANOVA (baseline x paced breathing). The effect of an anti-hyperventilation instruction was examined by analysis of variance for repeated measures separately for all outcomes of the study (PetCO2, respiratory rate, symptoms of hyperventilation, air hunger, and scores of dimensions of affective state) with moment of measurement (baseline x paced breathing) as a within-subject factor and type of instruction (control x anti-hyperventilation instruction) as a between-subject factor. Sex was added as a between-subject factor in the analysis of affect, because sex-related differences have been previously reported (Szulczewski and Rynkiewicz 2018). The influence of the anti-hyperventilation instruction on perceived task difficulty was examined using a two-way independent *t* test. The relationship between changes in symptoms of hyperventilation as well as the sensation of air hunger and drop in PetCO_2_ were examined by linear regression among individuals that experienced a decrease of PetCO_2_ between baseline and the breathing task. Changes in symptoms of hyperventilation and air hunger served as dependent variables and changes in PetCO_2_ as the independent variable. In order to investigate whether changes in PetCO_2_ were related to changes in affective state, linear regressions were performed with change in PetCO_2_ between baseline and breathing task as the independent variable and changes in affective state as dependent variables. Confidence intervals for the plots were calculated with the use of the data normalization procedure proposed by Morey (2008).

## Results

First, changes in respiratory rate were examined using a repeated measures ANOVA. Analysis showed that participants changed their breathing according to the breathing pacer, *F*(1, 43) = 208.81, *p* < 0.001, *η*^*2*^ = 0.83. Mean respiratory rates indicated that participants modified their respiratory rate in accordance with the breathing pacer (for means and standard deviations see Table 1). The mean respiratory rate was slightly higher than six breaths per minute; visual inspection of PetCO_2_ recordings indicated that this was caused by temporary increases in breathing frequency, likely due to compensatory reactions caused by hyperventilation and increased air hunger. One participant had a high mean respiratory rate (13.87 breaths per minute) during paced breathing due to massive hyperventilation that caused temporary increases in breathing frequency in the second half of the paced breathing task. Respiratory rates of other participants ranged from 6.04 to 9.39 breaths per minute.

**Table 1:** Means and standard deviations of the measures for the anti-hyperventilation instruction group and the control group

		Group	
		Instruction	Control
PetCO_2_ (mmHg)	Baseline	35.66 (5.33)	36.17 (4.49)
	Paced breathing	32.96 (5.71)	30.96 (5.40)
Respiratory rate (breaths/minute)	Baseline	16.76 (3.41)	15.44 (4.38)
	Paced breathing	6.88 (0.65)	7.04 (0.66)
Symptoms of hyperventilation	Baseline	0.51 (0.59)	0.49 (0.62)
	Paced breathing	0.69 (0.69)	1.12 (1.32)
Air hunger	Baseline	0.79 (1.08)	1.05 (1.28)
	Paced breathing	1.95 (1.51)	1.90 (1.65)
Task difficulty		2.21 (1.55)	2.05 (1.57)

*PetCO*_*2*_ pressure of end-tidal carbon dioxide

**Fig. 1:**
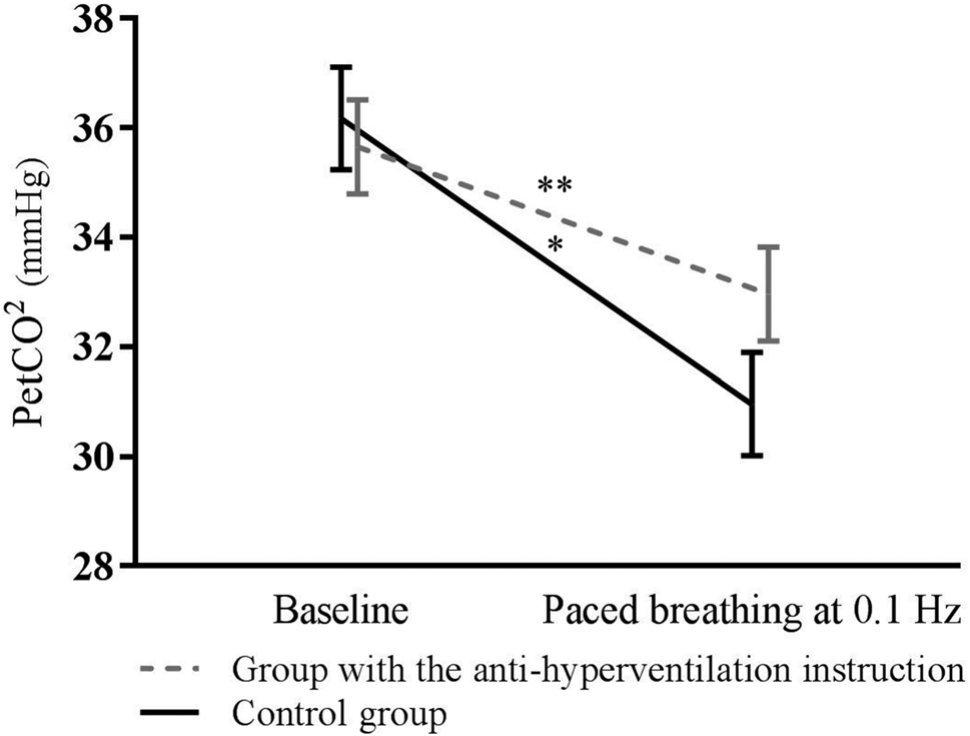
Changes in PetCO_2_ in the anti-hyperventilation instruction group and in the control group between baseline and paced breathing task as well as 95% Confidence intervals. *p < 0.05, **p < 0.001; the significance of the main effect of time of measurement is indicated above the lines and the significance of the interaction between group and time is indicated between the lines

**Fig. 2:**
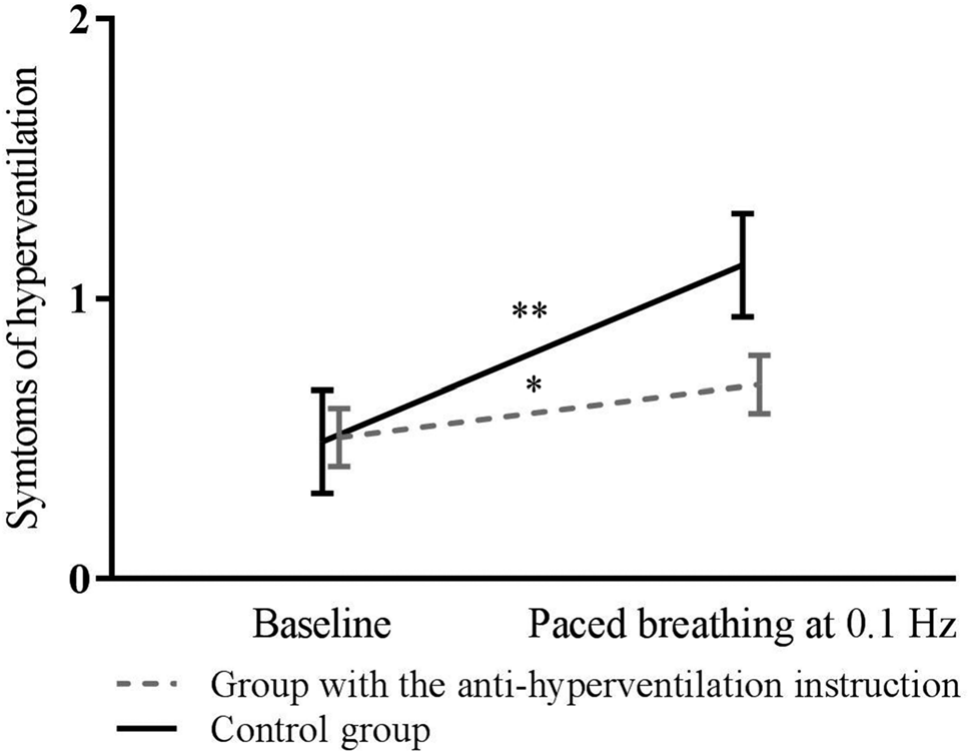
Changes in symptoms of hyperventilation in the anti-hyperventilation instruction group and in the control group between baseline and paced breathing task as well as 95% Confidence intervals. *p < 0.05, **p < 0.001; the significance of the main effect of time of measurement is indicated above the lines and the significance of the interaction between group and time is indicated between the lines

The effects of the anti-hyperventilation instruction were examined using a repeated measures ANOVA. PetCO_2_ was lower during breathing at 0.1 Hz than during baseline, *F*(1, 43) = 41.50, *p* < 0.001, *η*^*2*^ = 0.49, and PetCO_2_ decreased less in the group with the anti-hyperventilation instruction, *F*(1, 43) = 4.24, *p* < 0.05, *η*^*2*^ = 0.09. Furthermore, hyperventilation symptoms increased during paced breathing in comparison to baseline, *F*(1, 37) = 16.00, *p* < 0.001, *η*^*2*^ = 0.30, and they increased more in the control group than in the group with the anti-hyperventilation instruction, *F*(1, 37) = 4.63, *p* < 0.05, *η*^*2*^ = 0.11. Changes in PetCO_2_ and symptoms of hyperventilation are presented in Figs. 1 and 2. Air hunger increased during paced breathing in comparison to baseline, *F*(1, 37) = 23.00, *p* < 0.001, *η*^*2*^ = 0.38, and increase in air hunger sensation did not differ between conditions. Perceived task difficulty did not differ between groups (*p* > 0.05). Means and standard deviations of PetCO_2_, symptoms of hyperventilation, air hunger sensation, and task difficulty are presented in Table 1.

**Fig. 3:**
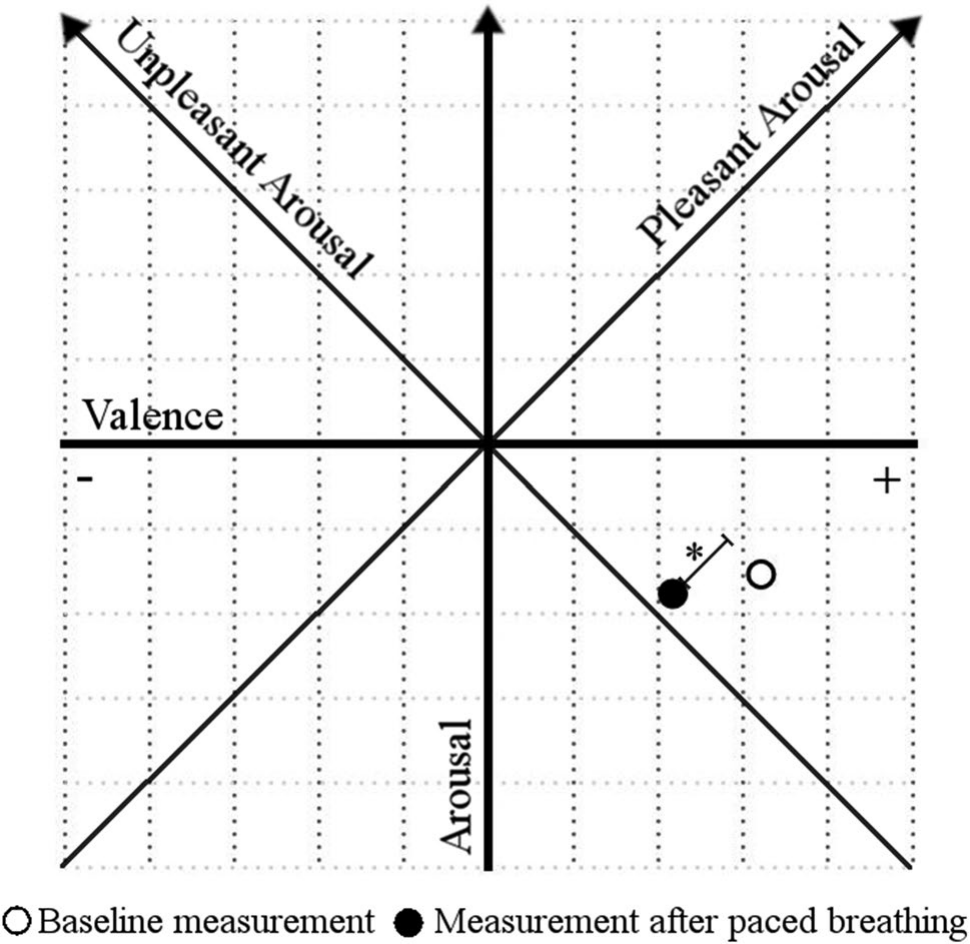
Affective state before and after the breathing task presented in two dimensional space with axes of general arousal and valence as well as axes of pleasant arousal and unpleasant arousal (at 45°). *p < 0.05; the only significant change was on the pleasant arousal axis

Affective state during the study is presented in Fig. 3. Only pleasant arousal (vitality) decreased significantly between baseline and measurement after the breathing task, *F*(1, 35) = 5.30, *p* < 0.05, *η*^*2*^ = 0.13. Scores of other dimensions of affective state did not differ significantly between baseline measurement and measurement after paced breathing and there were no effects of group and sex (all values of *p* > 0.05). Changes in PetCO_2_ between baseline and breathing task predicted changes in hyperventilation symptoms, *F*(1, 28) = 7.02, *p* < 0.05 with an *R*^*2*^ of 0.2, *β* = −0.09, *t* = -2.65, *p* < 0.05, but not changes in air hunger. Changes in PetCO_2_ did not predict changes in any dimension of affective state (all values of *p* > 0.05).

## Discussion

The main goal of the present study was to investigate the effects of a brief anti-hyperventilation instruction on PetCO_2_ and symptoms of hyperventilation during paced breathing at 0.1 Hz. Arterial level of CO_2_ has wide modulatory effects on multiple physiological processes and some of them are of particular interest for psychophysiology, such as, e.g., cardiovascular system activity. Furthermore, a drop in arterial CO_2_ tension produces a set of somatic and psychological symptoms. Because of this, it is desirable to avoid such a drop in both basic research and applied psychophysiology. The present study showed that a simple instruction to avoid excessively deep breathing and to breathe shallowly and naturally reduced both the drop in PetCO_2_ and self-reported somatic symptoms of hyperventilation.

Paced breathing at frequencies around 0.1 Hz is widely used in applied psychophysiology and basic research. Longer training of slow and shallow paced breathing with the guidance of a qualified coach or capnometry biofeedback removes the tendency to hyperventilate (Meuret et al. 2010; Meuret et al. 2008; Vaschillo et al. 2006). However, slow paced breathing is often used as a self-help method, for example several mobile apps implement it as a stress reduction tool (e.g. Paced Breathing, Trex LLC). Furthermore, it is often employed without training in research on the psychological and physiological effects of breathing frequency (e.g. Lin et al. 2014; Van Diest et al. 2014). Symptoms induced by hyperventilation can be aversive, which may discourage people from using paced breathing and may confound the results of basic research. A simple anti-hyperventilation instruction seems to be the first line of intervention to avoid hyperventilation. The present study suggests that a brief anti-hyperventilation instruction diminishes the tendency to hyperventilate during paced breathing at 0.1 Hz. Therefore, it may be used to decrease the confounding effects of a drop in PetCO_2_ and somatic symptoms of hyperventilation. However, despite a general decrease of drop in PetCO_2_ in the group with the anti-hyperventilation instruction, multiple participants nonetheless experienced a large drop in PetCO_2_. In the group with the instruction, 36.4% of participants had PetCO_2_ below 30 mmHg, in comparison to 43.5% in the control group. To better avoid hyperventilation, a more detailed instruction or guidance from a qualified coach equipped with a capnometer may be necessary. Furthermore, it is probable that longer training, even without any anti-hyperventilation instructions, will result in the adjustment of homeostatic mechanisms to paced breathing and the cessation of hyperventilation.

Paced breathing without an anti-hyperventilation instruction produced hyperventilation symptoms of modest intensity and there was no significant increase in hyperventilation symptoms in the group with the instruction. Previous studies that aimed to examine the effects of hyperventilation produced larger drops in PetCO_2_ (e.g. Wilhelm et al. 2001); drops of more than 50% from baseline values have been recommended to produce maximal symptoms of hyperventilation (Hornsveld et al., 1995). Drops in PetCO_2_ during breathing at 0.1 Hz observed in this and previous studies were modest (Lehrer et al. 1997; Szulczewski and Rynkiewicz 2018; Vaschillo et al. 2006). Therefore, hyperventilation during breathing at 0.1 Hz might be not deep enough to produce somatic symptoms. The present results showed that paced breathing at 0.1 Hz elicited symptoms of hyperventilation. However, the magnitude of the observed increase was low. In the control group, symptoms increased on average by 0.63 points (*SD* = 0.79) on a 7 point likert scale. In the group with the anti-hyperventilation instruction, there were no significant changes in symptoms of hyperventilation. The lack of relation between changes in PetCO_2_ and changes in affective state indicates that hyperventilation was not deep enough to alter affective state. Thus, the obtained results indicate that breathing at 0.1 Hz may produce minor hyperventilation symptoms but without changes in affective state and that the use of an anti-hyperventilation instruction reduced both drop in PetCO_2_ and symptoms of hyperventilation.

It was hypothesised that the instruction, which aimed to decrease hyperventilation, would increase the feeling of air hunger. The results did not support this hypothesis. In the present study, breathing at 0.1 Hz produced an increase in feeling of air hunger, but the between-group difference was not significant. It has been previously observed that breathing at 0.1 Hz increases breathing discomfort (dyspnea) among untrained individuals, and feeling of air hunger is one of the sensations of dyspnea (Allen and Friedman 2012). The observed air hunger sensation could be a result of hyperventilation, however, in contrast to other hyperventilation symptoms, drop in PetCO_2_ did not predict changes in air hunger sensation. Therefore, the increase of air hunger was likely caused by an increase of blood gas oscillations as a result of low respiratory rate. Arterial gas tension oscillates at the same frequency as breathing (Band et al. 1980; Semple 1984) and the amplitude of these oscillations increases when breathing frequency decreases (Takahashi et al. 1984). Increased amplitude of oscillations presumably leads to a transient increase in arterial CO_2_ at the end of exhalation, which elicits a feeling of air hunger. Accordingly, some participants reported after the experiment that they found the exhalation time too long. Interestingly, a study by Bernardi et al. (2001) showed that breathing at 0.1 Hz lessens increases in respiratory drive in response to hypoxia and hypercarbia. The present results suggest that this effect is not mediated by changes in air hunger sensation.

Because the feeling of air hunger has negative affective value, it may make breathing at 0.1 Hz more aversive, which is of importance for applied psychophysiology. The unpleasantness of breathing discomfort may be reduced by stimuli with positive emotional value (Von Leupoldt et al. 2006a, b; von Leupoldt et al. 2010); a study by Allen and Friedman (2012) showed the effectiveness of this method in reducing dyspnea during breathing at 0.1 Hz. Furthermore, the unpleasantness of air hunger can be modulated by cognitive factors, for example by modification of the interpretation of this interoceptive sensation (Bogaerts et al. 2005; Stegen et al. 2000). Air hunger during breathing at 0.1 Hz among most participants is not the result of hypoventilation but of a prolonged breathing cycle. This information can be used to change the cognitive interpretation of the sensation of air hunger and make it more tolerable.

Interestingly, the increased amplitude of arterial CO_2_ level oscillations and air hunger may have positive effects. One of the results of regular exposure to the sensation of air hunger during longer training of slow paced breathing may be increased tolerance to this dyspneic sensation, which can decrease the tendency to hyperventilate. Accordingly, training of slow breathing has been shown to increase tolerance of hypercarbia (Joshi et al. 1992; Miyamura et al. 2002; Stanescu et al. 1981). A recent study on hypoventilation therapy for panic disorder showed that exposure to the sensation of air hunger helps reduce panic symptoms (Meuret et al. 2018). Although increases in levels of arterial CO_2_ during slow paced breathing are smaller than during hypoventilation therapy, they might be sufficient to be involved in some psychophysiological effects of slow breathing training. Furthermore, it has been recently proposed that reduced CO_2_ sensitivity due to slow breathing training can have positive effects on attentional performance (Melnychuk et al. 2018).

Slow breathing is widely used and its anti-arousal effects are well established in applied psychophysiology. Previous studies have shown that slow paced breathing decreases psychological arousal when arousal has been increased by an experimental procedure such as, e.g., the threat of electric shock (Cappo and Holmes 1984; McCaul et al. 1979; Sakakibara and Hayano 1996). However, when negative affect was not increased, the reported results were less consistent, with some observing anti-arousal effects (Lin et al. 2014; Van Diest et al. 2014) but others not (Cappo and Holmes 1984; Stark et al. 2000; Szulczewski and Rynkiewicz 2018). The present results did not support anti-arousal effects of breathing at 0.1 Hz. Only pleasant arousal changed significantly between baseline and measurement after paced breathing. A comparable decrease of pleasant arousal was previously reported by Szulczewski and Rynkiewicz (2018) during breathing at 0.1 Hz and in a control group that breathed at 0.28 Hz. Therefore, this change presumably reflects boredom with the experimental procedure and is not related to breathing at 0.1 Hz. In the present study, participants were already in an affective state characterized by low arousal and pleasant valence before the breathing task (see Fig. 1). Therefore, the lack of effect on unpleasant arousal could be related to the low initial arousal. Another reason why affective state did not change might be the participants’ lack of experience with slow paced breathing. Presumably, longer training for this task is required to produce consistent anti-arousal effects.

The present and previous studies have shown that paced breathing produces hyperventilation among many participants (Anderson et al. 2009; Szulczewski and Rynkiewicz 2018). However, the issue of hyperventilation is often neglected in breathing techniques aimed at reducing psychophysiological arousal. Some methods even encourage deepening of breathing (e.g. deep breathing exercises; Syrjala and YI 2010). Indeed, slow breathing has to be deeper because it requires an increase in tidal volume to compensate for the decrease in respiratory rate. However, because overcompensation by breathing deeper than necessary often occurs, emphasis should not be put on deepening of breathing as a key element of the slow breathing exercise. Instead, an instruction to breathe shallow should be used, as is done in heart rate variability biofeedback (Lehrer et al. 2000) and Capnometry-Assisted Hypoventilation training (Meuret et al. 2008). Some breathing retraining methods assume that merely slowing down respiratory rate and increasing the use of the diaphragm without paying any attention to the depth of breathing has anti-hyperventilatory properties (Hazlett-Stevens and Craske 2009). Such methods have been tested for reducing hyperventilation among patients with panic disorder (for review see: Meuret et al. 2003). Present and previous findings indicate that merely slowing down respiratory rate is not an effective method of decreasing hyperventilation and that, during paced breathing exercises, attention should be paid to achieving adequate ventilation.

In summary, the present study showed that during breathing at 0.1 Hz, an instruction to avoid excessively deep breathing and to breathe shallowly and naturally decreased the drop in PetCO_2_ and reduced somatic symptoms of hyperventilation. The use of this instruction did not change the perceived difficulty of the task or affective state. Furthermore, the results indicate that breathing at 0.1 Hz may increase the feeling of air hunger among untrained participants. The present study suggests that a simple anti-hyperventilation instruction can be useful when a drop in arterial CO_2_ tension is not desirable, for example in basic research that employs paced breathing or when paced breathing is used as a relaxation technique.
