# Correction to: An Anti-hyperventilation Instruction Decreases the Drop in End-tidal CO_2_ and Symptoms of Hyperventilation During Breathing at 0.1 Hz

**DOI:** 10.1007/s10484-019-09441-3

**Published:** 2019-05-22

**Authors:** Mikołaj Tytus Szulczewski

**Affiliations:** Faculty of Psychology, University of Warsaw, Stawki 5/7, 00-183 Warsaw, Poland

## Correction to: Applied Psychophysiology and Biofeedback 10.1007/s10484-019-09438-y

The original version of this article unfortunately contained an error in abstract section. The number of participants should be “Forty-six participants” instead of “Thirty-six participants”.

The correct sentence is as follows:

Forty-six participants were randomly assigned to one of two groups: a group given an anti-hyperventilation instruction and a control group without such an instruction.

The original article has been corrected.

